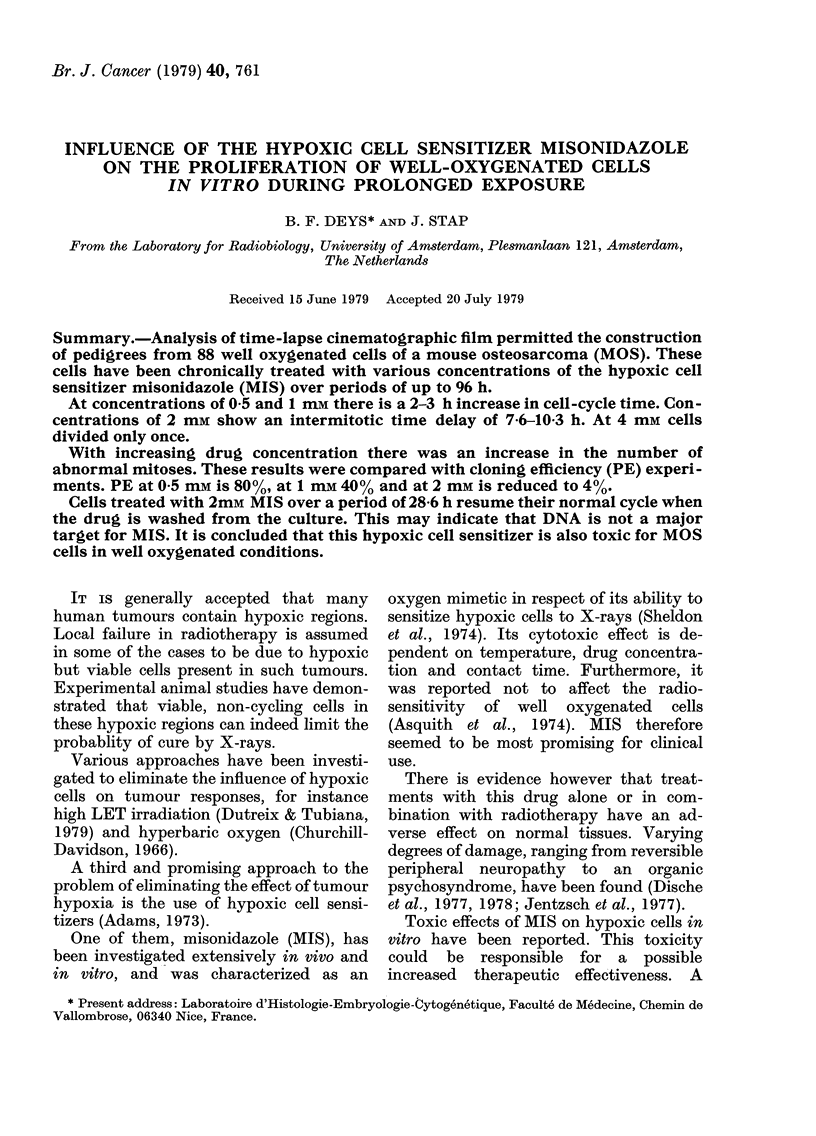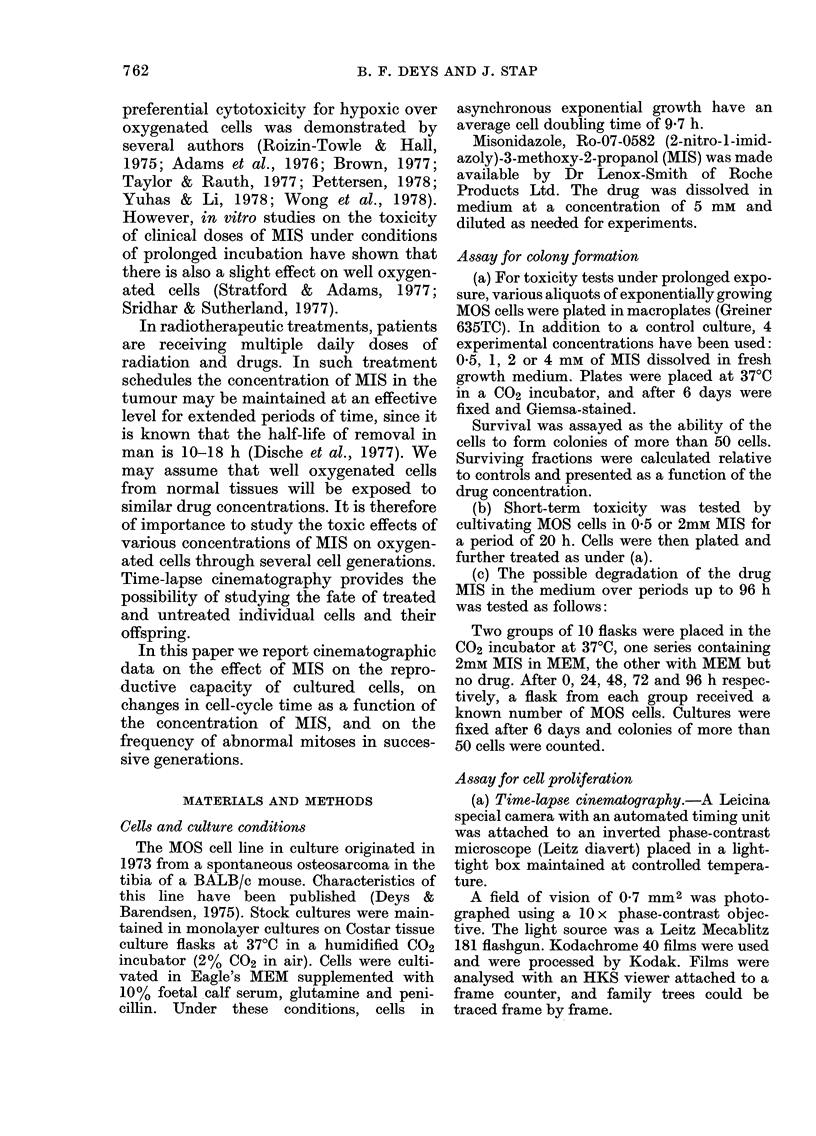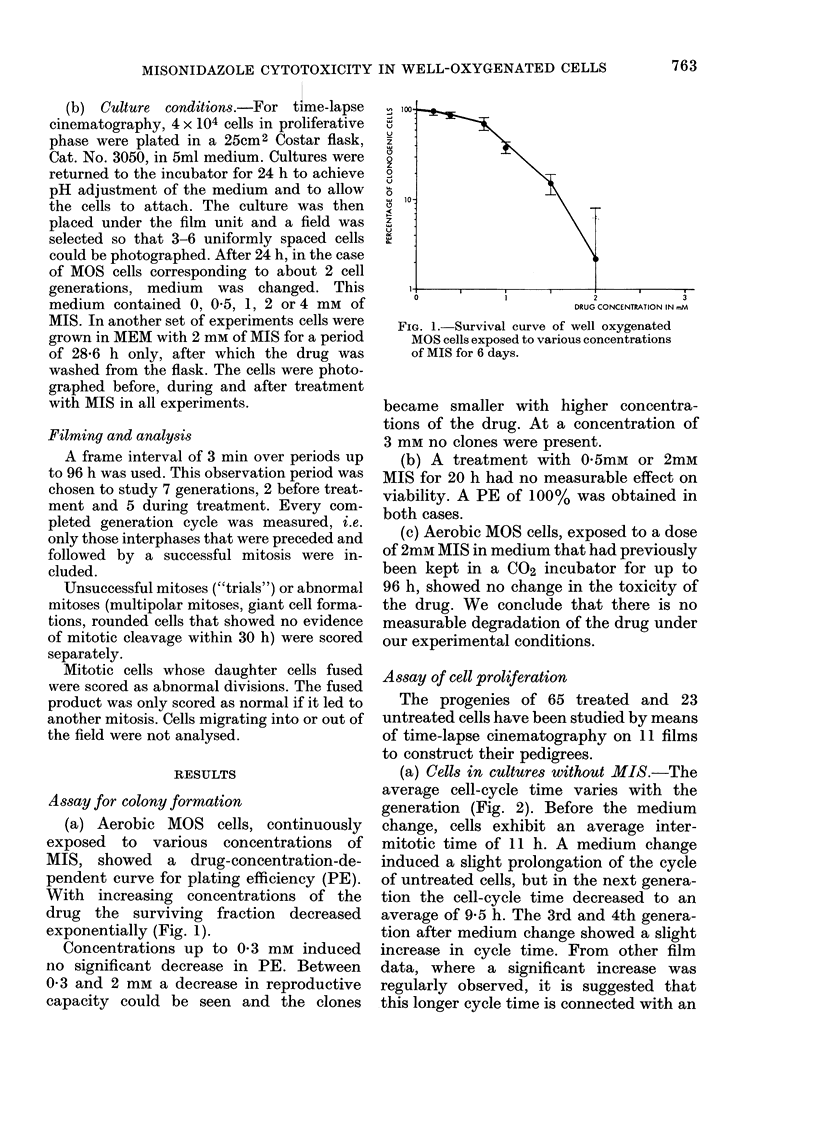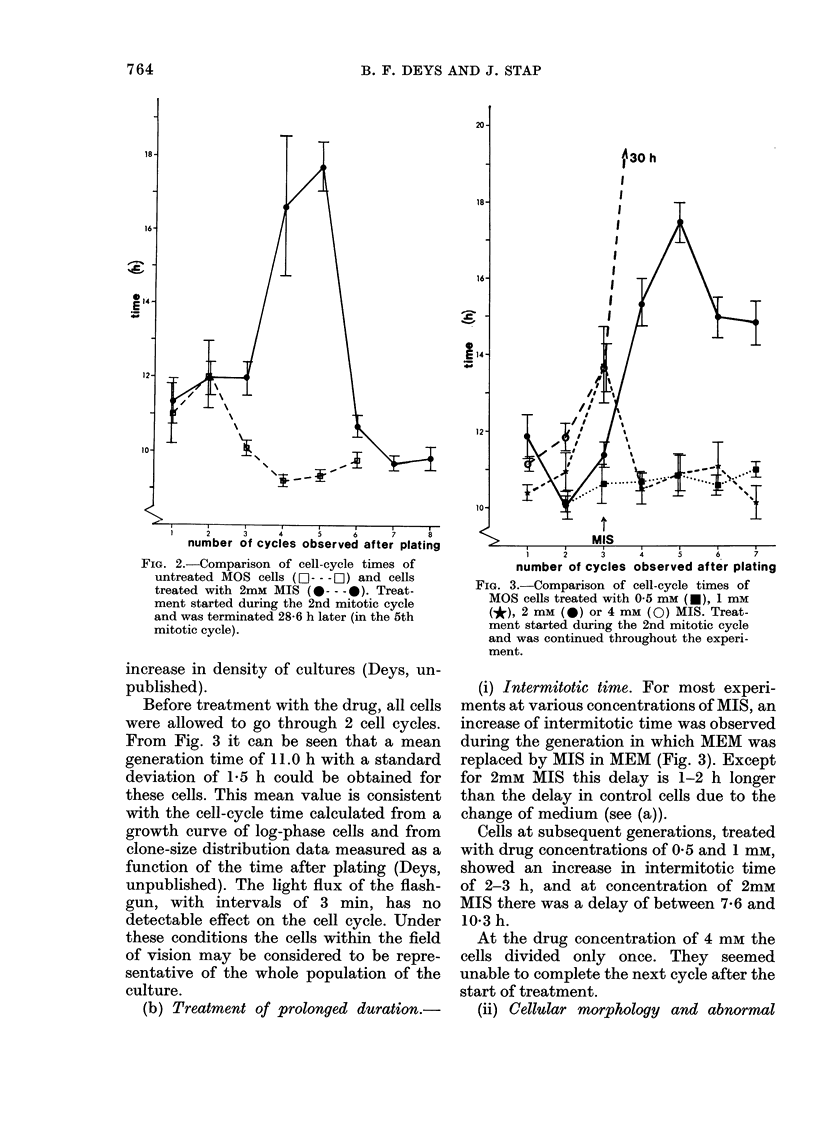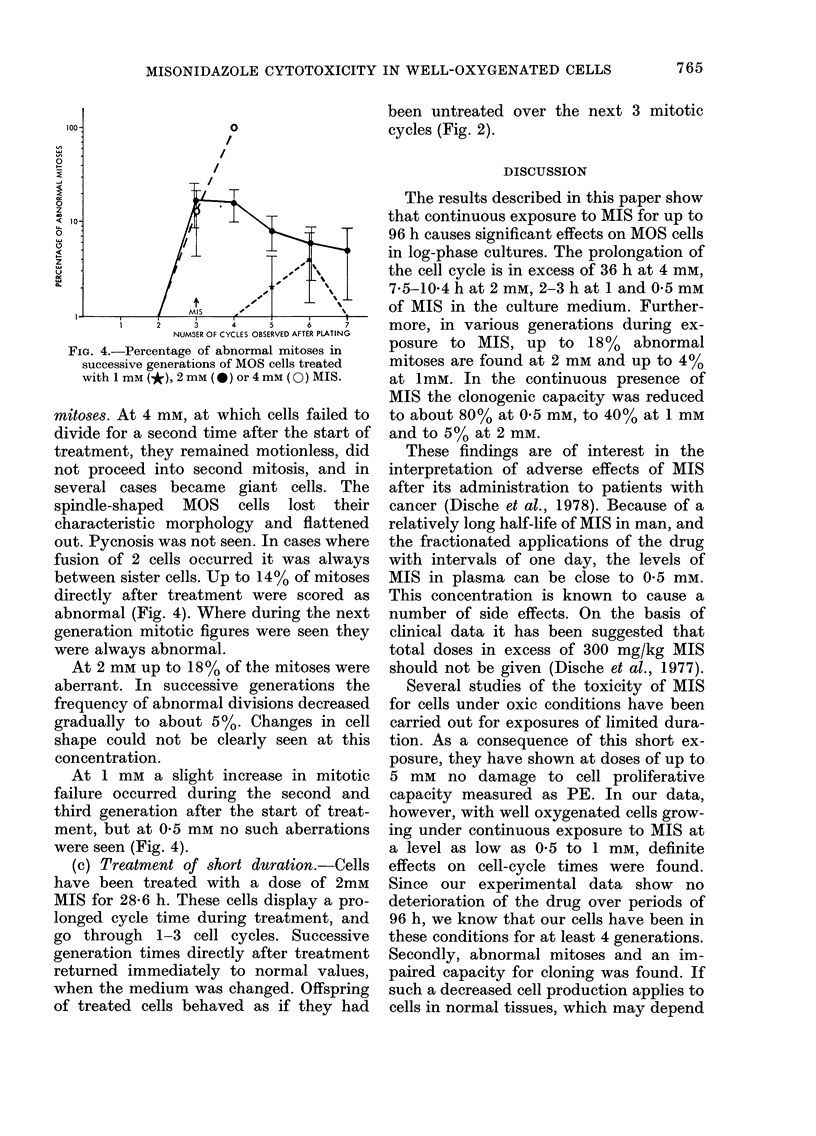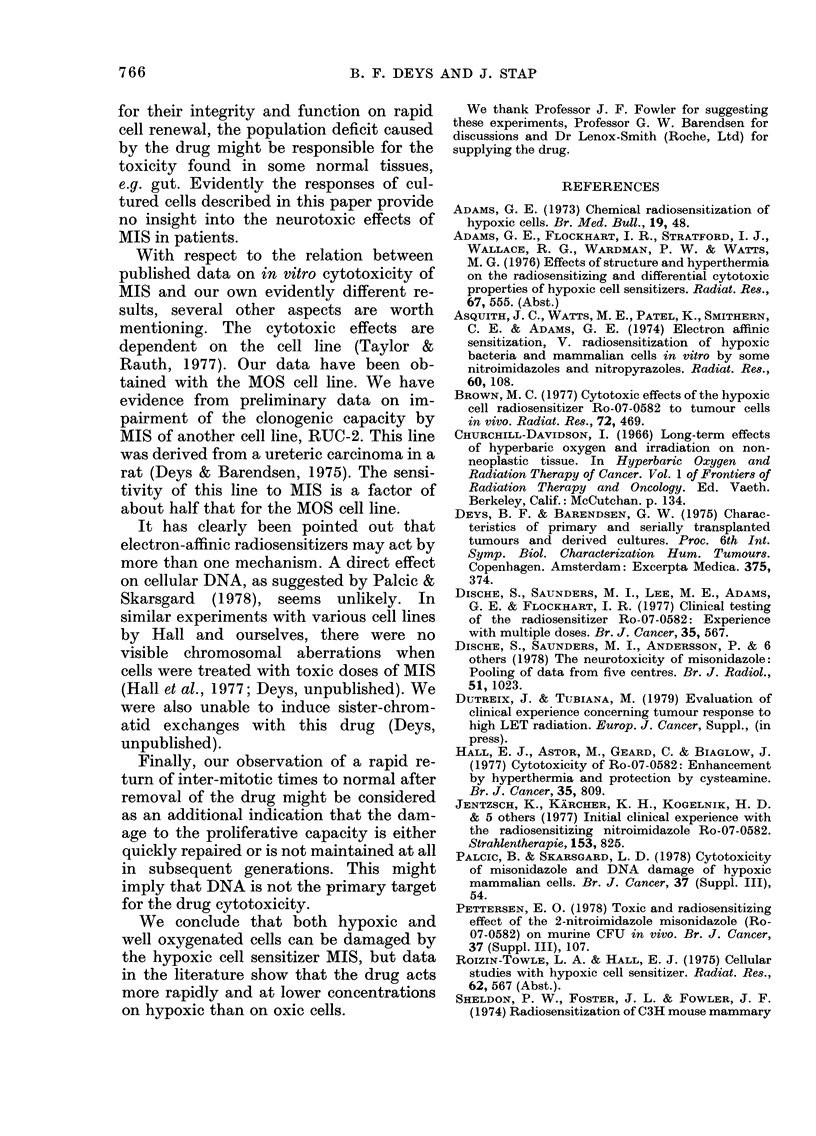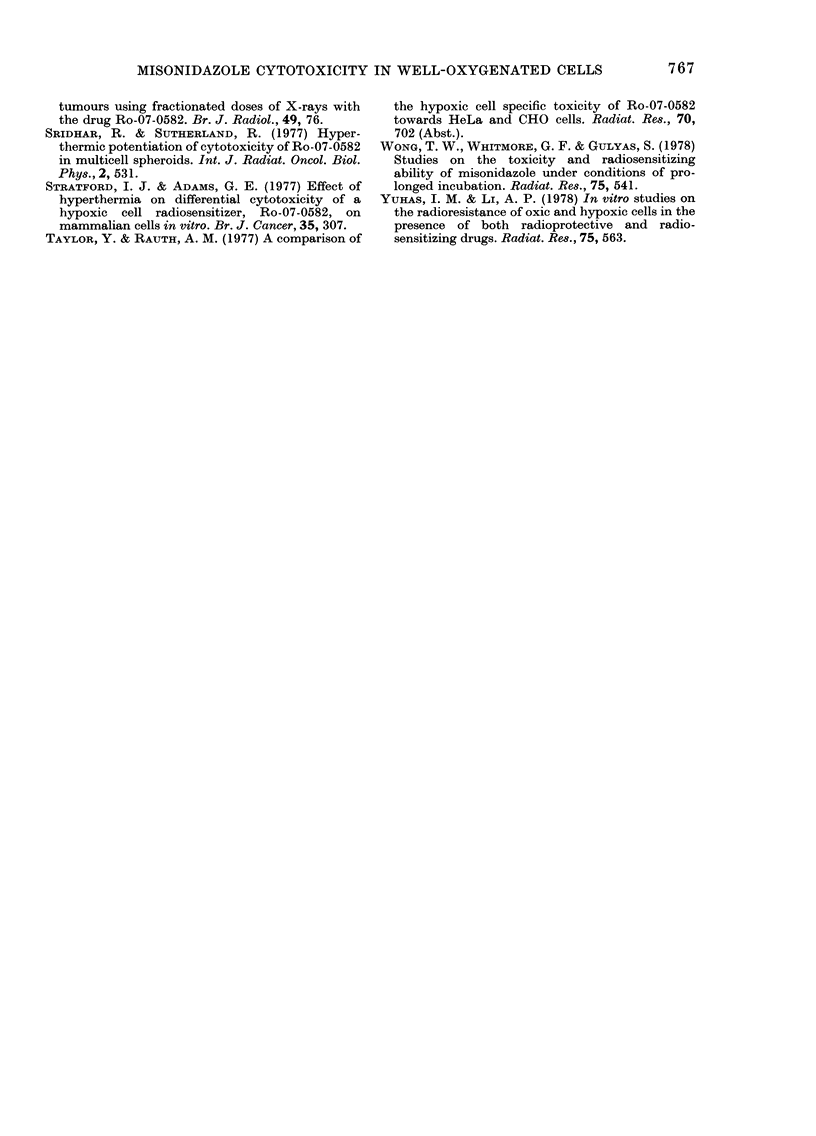# Influence of the hypoxic cell sensitizer misonidazole on the proliferation of well-oxygenated cells in vitro during prolonged exposure.

**DOI:** 10.1038/bjc.1979.258

**Published:** 1979-11

**Authors:** B. F. Deys, J. Stap

## Abstract

Analysis of time-lapse cinematographic film permitted the construction of pedigrees from 88 well oxygenated cells of a mouse osteosarcoma (MOS). These cells have been chronically treated with various concentrations of the hypoxic cell sensitizer misonidazole (MIS) over periods of up to 96 h. At concentrations of 0.5 and 7 mM there is a 2--3 h increase in cell-cycle time. Concentrations of 2 mM show an intermitotic time delay of 7.6--10.3 h. At 4 mM cells divided only once. With increasing drug concentration there was an increase in the number of abnormal mitoses. These results were compared with cloning efficiency (PE) experiments. PE at 0.5 mM is 80%, at 1 mM 40 and at 2 mM is reduced to 4%. Cells treated with 2mM MIS over a period of 28.6 h resume their normal cycle when the drug is washed from the culture. This may indicate that DNA is not a major target for MIS. It is concluded that this hypoxic cell sensitizer is also toxic for MOS cells in well oxygenated conditions.


*Br. J. Cancer* (1979) **40**, 761

### INFLUENCE OF THE HYPOXIC CELL SENSITIZER MISONIDAZOLE ON THE PROLIFERATION OF WELL-OXYGENATED CELLS *IN VITRO* DURING PROLONGED EXPOSURE

B. F. DEYS* AND J. STAP

*From the Laboratory for Radiobiology, University of Amsterdam, Plesmanlaan* 121, *Amsterdam, The Netherlands*

Received 15 June 1979 Accepted 20 July 1979

**Summary.—Analysis of time-lapse cinematographic film permitted the construction of pedigrees from 88 well oxygenated cells of a mouse osteosarcoma (MOS). These cells have been chronically treated with various concentrations of the hypoxic cell sensitizer misonidazole (MIS) over periods of up to 96 h.**

**At concentrations of 0·5 and 1 mM there is a 2–3 h increase in cell-cycle time. Concentrations of 2 mM show an intermitotic time delay of 7·6–10·3 h. At 4 mM cells divided only once.**

**With increasing drug concentration there was an increase in the number of abnormal mitoses. These results were compared with cloning efficiency (PE) experiments. PE at 0·5 mM is 80%, at 1 mM 40% and at 2 mM is reduced to 4%.**

**Cells treated with 2mM MIS over a period of 28·6 h resume their normal cycle when the drug is washed from the culture. This may indicate that DNA is not a major target for MIS. It is concluded that this hypoxic cell sensitizer is also toxic for MOS cells in well oxygenated conditions.**

IT IS generally accepted that many human tumours contain hypoxic regions. Local failure in radiotherapy is assumed in some of the cases to be due to hypoxic but viable cells present in such tumours. Experimental animal studies have demonstrated that viable, non-cycling cells in these hypoxic regions can indeed limit the probablity of cure by X-rays.

Various approaches have been investigated to eliminate the influence of hypoxic cells on tumour responses, for instance high LET irradiation (Dutreix & Tubiana, 1979) and hyperbaric oxygen (Churchill-Davidson, 1966).

A third and promising approach to the problem of eliminating the effect of tumour hypoxia is the use of hypoxic cell sensitizers (Adams, 1973).

One of them, misonidazole (MIS), has been investigated extensively *in vivo* and *in vitro*, and was characterized as an oxygen mimetic in respect of its ability to sensitize hypoxic cells to X-rays (Sheldon *et al.*, 1974). Its cytotoxic effect is dependent on temperature, drug concentration and contact time. Furthermore, it was reported not to affect the radiosensitivity of well oxygenated cells (Asquith *et al.*, 1974). MIS therefore seemed to be most promising for clinical use.

There is evidence however that treatments with this drug alone or in combination with radiotherapy have an adverse effect on normal tissues. Varying degrees of damage, ranging from reversible peripheral neuropathy to an organic psychosyndrome, have been found (Dische *et al.*, 1977, 1978; Jentzsch *et al.*, 1977).

Toxic effects of MIS on hypoxic cells *in vitro* have been reported. This toxicity could be responsible for a possible increased therapeutic effectiveness. A

* Present address: Laboratoire d'Histologie-Embryologie-Cytogénétique, Faculté de Médecine, Chemin de Vallombrose, 06340 Nice, France.

preferential cytotoxicity for hypoxic over oxygenated cells was demonstrated by several authors (Roizin-Towle & Hall, 1975; Adams *et al.*, 1976; Brown, 1977; Taylor & Rauth, 1977; Pettersen, 1978; Yuhas & Li, 1978; Wong *et al.*, 1978). However, *in vitro* studies on the toxicity of clinical doses of MIS under conditions of prolonged incubation have shown that there is also a slight effect on well oxygenated cells (Stratford & Adams, 1977; Sridhar & Sutherland, 1977).

In radiotherapeutic treatments, patients are receiving multiple daily doses of radiation and drugs. In such treatment schedules the concentration of MIS in the tumour may be maintained at an effective level for extended periods of time, since it is known that the half-life of removal in man is 10–18 h (Dische *et al.*, 1977). We may assume that well oxygenated cells from normal tissues will be exposed to similar drug concentrations. It is therefore of importance to study the toxic effects of various concentrations of MIS on oxygenated cells through several cell generations. Time-lapse cinematography provides the possibility of studying the fate of treated and untreated individual cells and their offspring.

In this paper we report cinematographic data on the effect of MIS on the reproductive capacity of cultured cells, on changes in cell-cycle time as a function of the concentration of MIS, and on the frequency of abnormal mitoses in successive generations.

### MATERIALS AND METHODS

### *Cells and culture conditions*

The MOS cell line in culture originated in 1973 from a spontaneous osteosarcoma in the tibia of a BALB/c mouse. Characteristics of this line have been published (Deys & Barendsen, 1975). Stock cultures were maintained in monolayer cultures on Costar tissue culture flasks at 37°C in a humidified $CO_2$ incubator (2% $CO_2$ in air). Cells were cultivated in Eagle's MEM supplemented with 10% foetal calf serum, glutamine and penicillin. Under these conditions, cells in asynchronous exponential growth have an average cell doubling time of 9·7 h.

Misonidazole, Ro-07-0582 (2-nitro-1-imidazoly)-3-methoxy-2-propanol (MIS) was made available by Dr Lenox-Smith of Roche Products Ltd. The drug was dissolved in medium at a concentration of 5 mM and diluted as needed for experiments.

### *Assay for colony formation*

(a) For toxicity tests under prolonged exposure, various aliquots of exponentially growing MOS cells were plated in macroplates (Greiner 635TC). In addition to a control culture, 4 experimental concentrations have been used: 0·5, 1, 2 or 4 mM of MIS dissolved in fresh growth medium. Plates were placed at 37°C in a $CO_2$ incubator, and after 6 days were fixed and Giemsa-stained.

Survival was assayed as the ability of the cells to form colonies of more than 50 cells. Surviving fractions were calculated relative to controls and presented as a function of the drug concentration.

(b) Short-term toxicity was tested by cultivating MOS cells in 0·5 or 2mM MIS for a period of 20 h. Cells were then plated and further treated as under (a).

(c) The possible degradation of the drug MIS in the medium over periods up to 96 h was tested as follows:

Two groups of 10 flasks were placed in the $CO_2$ incubator at 37°C, one series containing 2mM MIS in MEM, the other with MEM but no drug. After 0, 24, 48, 72 and 96 h respectively, a flask from each group received a known number of MOS cells. Cultures were fixed after 6 days and colonies of more than 50 cells were counted.

### *Assay for cell proliferation*

(a) *Time-lapse cinematography.*—A Leicina special camera with an automated timing unit was attached to an inverted phase-contrast microscope (Leitz diavert) placed in a light-tight box maintained at controlled temperature.

A field of vision of 0·7 mm$^2$ was photographed using a 10× phase-contrast objective. The light source was a Leitz Mecablitz 181 flashgun. Kodachrome 40 films were used and were processed by Kodak. Films were analysed with an HKS viewer attached to a frame counter, and family trees could be traced frame by frame.

(b) *Culture conditions.*—For time-lapse cinematography, $4 \times 10^4$ cells in proliferative phase were plated in a $25cm^2$ Costar flask, Cat. No. 3050, in 5ml medium. Cultures were returned to the incubator for 24 h to achieve pH adjustment of the medium and to allow the cells to attach. The culture was then placed under the film unit and a field was selected so that 3–6 uniformly spaced cells could be photographed. After 24 h, in the case of MOS cells corresponding to about 2 cell generations, medium was changed. This medium contained 0, 0·5, 1, 2 or 4 mM of MIS. In another set of experiments cells were grown in MEM with 2 mM of MIS for a period of 28·6 h only, after which the drug was washed from the flask. The cells were photographed before, during and after treatment with MIS in all experiments.

*Filming and analysis*

A frame interval of 3 min over periods up to 96 h was used. This observation period was chosen to study 7 generations, 2 before treatment and 5 during treatment. Every completed generation cycle was measured, *i.e.* only those interphases that were preceded and followed by a successful mitosis were included.

Unsuccessful mitoses ("trials") or abnormal mitoses (multipolar mitoses, giant cell formations, rounded cells that showed no evidence of mitotic cleavage within 30 h) were scored separately.

Mitotic cells whose daughter cells fused were scored as abnormal divisions. The fused product was only scored as normal if it led to another mitosis. Cells migrating into or out of the field were not analysed.

RESULTS

*Assay for colony formation*

(a) Aerobic MOS cells, continuously exposed to various concentrations of MIS, showed a drug-concentration-dependent curve for plating efficiency (PE). With increasing concentrations of the drug the surviving fraction decreased exponentially (Fig. 1).

Concentrations up to 0·3 mM induced no significant decrease in PE. Between 0·3 and 2 mM a decrease in reproductive capacity could be seen and the clones

FIG. 1.—Survival curve of well oxygenated MOS cells exposed to various concentrations of MIS for 6 days.

became smaller with higher concentrations of the drug. At a concentration of 3 mM no clones were present.

(b) A treatment with 0·5mM or 2mM MIS for 20 h had no measurable effect on viability. A PE of 100% was obtained in both cases.

(c) Aerobic MOS cells, exposed to a dose of 2mM MIS in medium that had previously been kept in a $CO_2$ incubator for up to 96 h, showed no change in the toxicity of the drug. We conclude that there is no measurable degradation of the drug under our experimental conditions.

*Assay of cell proliferation*

The progenies of 65 treated and 23 untreated cells have been studied by means of time-lapse cinematography on 11 films to construct their pedigrees.

(a) *Cells in cultures without MIS.*—The average cell-cycle time varies with the generation (Fig. 2). Before the medium change, cells exhibit an average intermitotic time of 11 h. A medium change induced a slight prolongation of the cycle of untreated cells, but in the next generation the cell-cycle time decreased to an average of 9·5 h. The 3rd and 4th generation after medium change showed a slight increase in cycle time. From other film data, where a significant increase was regularly observed, it is suggested that this longer cycle time is connected with an

FIG. 2.—Comparison of cell-cycle times of untreated MOS cells (□- - -□) and cells treated with 2mM MIS (●- - -●). Treatment started during the 2nd mitotic cycle and was terminated 28·6 h later (in the 5th mitotic cycle).

FIG. 3.—Comparison of cell-cycle times of MOS cells treated with 0·5 mM (■), 1 mM (★), 2 mM (●) or 4 mM (○) MIS. Treatment started during the 2nd mitotic cycle and was continued throughout the experiment.

increase in density of cultures (Deys, unpublished).

Before treatment with the drug, all cells were allowed to go through 2 cell cycles. From Fig. 3 it can be seen that a mean generation time of 11.0 h with a standard deviation of 1·5 h could be obtained for these cells. This mean value is consistent with the cell-cycle time calculated from a growth curve of log-phase cells and from clone-size distribution data measured as a function of the time after plating (Deys, unpublished). The light flux of the flash-gun, with intervals of 3 min, has no detectable effect on the cell cycle. Under these conditions the cells within the field of vision may be considered to be representative of the whole population of the culture.

(b) *Treatment of prolonged duration.*—

(i) *Intermitotic time.* For most experiments at various concentrations of MIS, an increase of intermitotic time was observed during the generation in which MEM was replaced by MIS in MEM (Fig. 3). Except for 2mM MIS this delay is 1–2 h longer than the delay in control cells due to the change of medium (see (a)).

Cells at subsequent generations, treated with drug concentrations of 0·5 and 1 mM, showed an increase in intermitotic time of 2–3 h, and at concentration of 2mM MIS there was a delay of between 7·6 and 10·3 h.

At the drug concentration of 4 mM the cells divided only once. They seemed unable to complete the next cycle after the start of treatment.

(ii) *Cellular morphology and abnormal*

FIG. 4.—Percentage of abnormal mitoses in successive generations of MOS cells treated with 1 mM (★), 2 mM (●) or 4 mM (○) MIS.

*mitoses*. At 4 mM, at which cells failed to divide for a second time after the start of treatment, they remained motionless, did not proceed into second mitosis, and in several cases became giant cells. The spindle-shaped MOS cells lost their characteristic morphology and flattened out. Pycnosis was not seen. In cases where fusion of 2 cells occurred it was always between sister cells. Up to 14% of mitoses directly after treatment were scored as abnormal (Fig. 4). Where during the next generation mitotic figures were seen they were always abnormal.

At 2 mM up to 18% of the mitoses were aberrant. In successive generations the frequency of abnormal divisions decreased gradually to about 5%. Changes in cell shape could not be clearly seen at this concentration.

At 1 mM a slight increase in mitotic failure occurred during the second and third generation after the start of treatment, but at 0·5 mM no such aberrations were seen (Fig. 4).

(c) *Treatment of short duration*.—Cells have been treated with a dose of 2mM MIS for 28·6 h. These cells display a prolonged cycle time during treatment, and go through 1–3 cell cycles. Successive generation times directly after treatment returned immediately to normal values, when the medium was changed. Offspring of treated cells behaved as if they had been untreated over the next 3 mitotic cycles (Fig. 2).

### DISCUSSION

The results described in this paper show that continuous exposure to MIS for up to 96 h causes significant effects on MOS cells in log-phase cultures. The prolongation of the cell cycle is in excess of 36 h at 4 mM, 7·5–10·4 h at 2 mM, 2–3 h at 1 and 0·5 mM of MIS in the culture medium. Furthermore, in various generations during exposure to MIS, up to 18% abnormal mitoses are found at 2 mM and up to 4% at 1mM. In the continuous presence of MIS the clonogenic capacity was reduced to about 80% at 0·5 mM, to 40% at 1 mM and to 5% at 2 mM.

These findings are of interest in the interpretation of adverse effects of MIS after its administration to patients with cancer (Dische *et al.*, 1978). Because of a relatively long half-life of MIS in man, and the fractionated applications of the drug with intervals of one day, the levels of MIS in plasma can be close to 0·5 mM. This concentration is known to cause a number of side effects. On the basis of clinical data it has been suggested that total doses in excess of 300 mg/kg MIS should not be given (Dische *et al.*, 1977).

Several studies of the toxicity of MIS for cells under oxic conditions have been carried out for exposures of limited duration. As a consequence of this short exposure, they have shown at doses of up to 5 mM no damage to cell proliferative capacity measured as PE. In our data, however, with well oxygenated cells growing under continuous exposure to MIS at a level as low as 0·5 to 1 mM, definite effects on cell-cycle times were found. Since our experimental data show no deterioration of the drug over periods of 96 h, we know that our cells have been in these conditions for at least 4 generations. Secondly, abnormal mitoses and an impaired capacity for cloning was found. If such a decreased cell production applies to cells in normal tissues, which may depend for their integrity and function on rapid cell renewal, the population deficit caused by the drug might be responsible for the toxicity found in some normal tissues, *e.g.* gut. Evidently the responses of cultured cells described in this paper provide no insight into the neurotoxic effects of MIS in patients.

With respect to the relation between published data on *in vitro* cytotoxicity of MIS and our own evidently different results, several other aspects are worth mentioning. The cytotoxic effects are dependent on the cell line (Taylor & Rauth, 1977). Our data have been obtained with the MOS cell line. We have evidence from preliminary data on impairment of the clonogenic capacity by MIS of another cell line, RUC-2. This line was derived from a ureteric carcinoma in a rat (Deys & Barendsen, 1975). The sensitivity of this line to MIS is a factor of about half that for the MOS cell line.

It has clearly been pointed out that electron-affinic radiosensitizers may act by more than one mechanism. A direct effect on cellular DNA, as suggested by Palcic & Skarsgard (1978), seems unlikely. In similar experiments with various cell lines by Hall and ourselves, there were no visible chromosomal aberrations when cells were treated with toxic doses of MIS (Hall *et al.*, 1977; Deys, unpublished). We were also unable to induce sister-chromatid exchanges with this drug (Deys, unpublished).

Finally, our observation of a rapid return of inter-mitotic times to normal after removal of the drug might be considered as an additional indication that the damage to the proliferative capacity is either quickly repaired or is not maintained at all in subsequent generations. This might imply that DNA is not the primary target for the drug cytotoxicity.

We conclude that both hypoxic and well oxygenated cells can be damaged by the hypoxic cell sensitizer MIS, but data in the literature show that the drug acts more rapidly and at lower concentrations on hypoxic than on oxic cells.

We thank Professor J. F. Fowler for suggesting these experiments, Professor G. W. Barendsen for discussions and Dr Lenox-Smith (Roche, Ltd) for supplying the drug.

### REFERENCES

ADAMS, G. E. (1973) Chemical radiosensitization of hypoxic cells. *Br. Med. Bull.*, **19**, 48.

ADAMS, G. E., FLOCKHART, I. R., STRATFORD, I. J., WALLACE, R. G., WARDMAN, P. W. & WATTS, M. G. (1976) Effects of structure and hyperthermia on the radiosensitizing and differential cytotoxic properties of hypoxic cell sensitizers. *Radiat. Res.*, **67**, 555. (Abst.)

ASQUITH, J. C., WATTS, M. E., PATEL, K., SMITHERN, C. E. & ADAMS, G. E. (1974) Electron affinic sensitization, V. radiosensitization of hypoxic bacteria and mammalian cells *in vitro* by some nitroimidazoles and nitropyrazoles. *Radiat. Res.*, **60**, 108.

BROWN, M. C. (1977) Cytotoxic effects of the hypoxic cell radiosensitizer Ro-07-0582 to tumour cells *in vivo*. *Radiat. Res.*, **72**, 469.

CHURCHILL-DAVIDSON, I. (1966) Long-term effects of hyperbaric oxygen and irradiation on non-neoplastic tissue. In *Hyperbaric Oxygen and Radiation Therapy of Cancer. Vol.* 1 *of Frontiers of Radiation Therapy and Oncology*. Ed. Vaeth. Berkeley, Calif.: McCutchan. p. 134.

DEYS, B. F. & BARENDSEN, G. W. (1975) Characteristics of primary and serially transplanted tumours and derived cultures. *Proc. 6th Int. Symp. Biol. Characterization Hum. Tumours*. Copenhagen. Amsterdam: Excerpta Medica. **375**, 374.

DISCHE, S., SAUNDERS, M. I., LEE, M. E., ADAMS, G. E. & FLOCKHART, I. R. (1977) Clinical testing of the radiosensitizer Ro-07-0582: Experience with multiple doses. *Br. J. Cancer*, **35**, 567.

DISCHE, S., SAUNDERS, M. I., ANDERSSON, P. & 6 others (1978) The neurotoxicity of misonidazole: Pooling of data from five centres. *Br. J. Radiol.*, **51**, 1023.

DUTREIX, J. & TUBIANA, M. (1979) Evaluation of clinical experience concerning tumour response to high LET radiation. *Europ. J. Cancer*, Suppl., (in press).

HALL, E. J., ASTOR, M., GEARD, C. & BIAGLOW, J. (1977) Cytotoxicity of Ro-07-0582: Enhancement by hyperthermia and protection by cysteamine. *Br. J. Cancer*, **35**, 809.

JENTZSCH, K., KÄRCHER, K. H., KOGELNIK, H. D. & 5 others (1977) Initial clinical experience with the radiosensitizing nitroimidazole Ro-07-0582. *Strahlentherapie*, **153**, 825.

PALCIC, B. & SKARSGARD, L. D. (1978) Cytotoxicity of misonidazole and DNA damage of hypoxic mammalian cells. *Br. J. Cancer*, **37** (Suppl. III), 54.

PETTERSEN, E. O. (1978) Toxic and radiosensitizing effect of the 2-nitroimidazole misonidazole (Ro-07-0582) on murine CFU *in vivo*. *Br. J. Cancer*, **37** (Suppl. III), 107.

ROIZIN-TOWLE, L. A. & HALL, E. J. (1975) Cellular studies with hypoxic cell sensitizer. *Radiat. Res.*, **62**, 567 (Abst.).

SHELDON, P. W., FOSTER, J. L. & FOWLER, J. F. (1974) Radiosensitization of C3H mouse mammary tumours using fractionated doses of X-rays with the drug Ro-07-0582. *Br. J. Radiol.*, **49**, 76.

SRIDHAR, R. & SUTHERLAND, R. (1977) Hyperthermic potentiation of cytotoxicity of Ro-07-0582 in multicell spheroids. *Int. J. Radiat. Oncol. Biol. Phys.*, **2**, 531.

STRATFORD, I. J. & ADAMS, G. E. (1977) Effect of hyperthermia on differential cytotoxicity of a hypoxic cell radiosensitizer, Ro-07-0582, on mammalian cells *in vitro*. *Br. J. Cancer*, **35**, 307.

TAYLOR, Y. & RAUTH, A. M. (1977) A comparison of the hypoxic cell specific toxicity of Ro-07-0582 towards HeLa and CHO cells. *Radiat. Res.*, **70**, 702 (Abst.).

WONG, T. W., WHITMORE, G. F. & GULYAS, S. (1978) Studies on the toxicity and radiosensitizing ability of misonidazole under conditions of prolonged incubation. *Radiat. Res.*, **75**, 541.

YUHAS, I. M. & LI, A. P. (1978) *In vitro* studies on the radioresistance of oxic and hypoxic cells in the presence of both radioprotective and radiosensitizing drugs. *Radiat. Res.*, **75**, 563.